# Impact of the Extended Digital Eco-Dynamic on Innovation Performance: An Empirical Study on Small E-Businesses in Indonesia

**DOI:** 10.3389/fpsyg.2022.864530

**Published:** 2022-03-30

**Authors:** Yuniarty Yuniarty, Idris Gautama So, Sri Bramantoro Abdinagoro, Mohammad Hamsal

**Affiliations:** Management Department, BINUS Business School Doctor of Research in Management, Bina Nusantara University, Jakarta, Indonesia

**Keywords:** rich, innovation, performance, Indonesia, e-business, COVID-19 pandemic

## Abstract

This study will answer the factors that influence the innovation performance of small e-businesses in Indonesia during the COVID-19 pandemic. The results of this study are expected to contribute to the development of innovation theory by enriching knowledge in the field of management science in general, especially entrepreneurship theory, especially those related to innovation performance, IT ambidexterity, dynamic capability, environmental uncertainty, and Resource-Induced Coping Heuristic (RICH). This study proposes novelty by examining the effect of acquiring, developing, and protecting resources as dimensions of RICH on innovation performance in turbulent conditions due to the COVID-19 pandemic. This theoretical aspect forms the basis for further research that will develop into a broader scope related to innovation performance in entrepreneurship in Indonesia. This fundamental research uses a questionnaire as a data collection tool tested for validity and reliability before being tested empirically using structural equation modeling partial least squares. Research shows that among the dimensions of RICH, protecting resources is the most influential on innovation performance, followed by acquiring resources and developing resources. Dynamic capability positively affects innovation performance. Environmental uncertainty positively affects dynamic capability. Environmental uncertainty positively affects innovation performance. IT capability positively affects dynamic capability. IT capability positively affects innovation performance. The development of small e-businesses needs to get significant attention. Small e-businesses need to develop mutually beneficial business partnerships and improve the quality of their human resources.

## Introduction

The increase in the number of online shop members has made the value of e-commerce transactions in Indonesia increase; wherein 2018, it reached Rp. 77.766 trillion. This picture skyrocketed 151%, contrasted to the previous year, reaching Rp 30, 942 trillion (BPPK, 2021). This proves that business through e-commerce is increasingly promising. Consumers have begun to rely on e-commerce sites to buy various items, particularly those that are difficult to obtain in physical stores, which has resulted in the rapid rise of the e-commerce industry. Moreover, as the number of Internet users grows year after year (KOMINFO, 2021), e-commerce can be an excellent opportunity for MSME players to develop their business and allow MSMEs to do marketing with the aim of global markets so that they have the prospect to penetrate exports (KOMMINFO, 2018; KOMINFO, 2021).

This e-commerce opportunity must also be utilized by various industries, including the creative industry in Indonesia. The creative economy is described as the embodiment of added value created by human ingenuity based on cultural legacy, science, and technology (BEKRAF, 2020). The top three creative industries in Indonesia are the culinary, fashion, and craft sectors. This study will contribute to defining factors to improve these sub-sectors’ innovation performance. Increasing the competitiveness of the creative industry through optimizing the use of technology in its business activities, developing creativity and creative industry institutions, and becoming the focus of developing the creative economy sub-sector (KEMENPAREKRAF, 2015).

In 2018, the share of creative economy workers was 18.21%, and the order of sub-sectors based on the most significant number of workers was the culinary sub-sector (BEKRAF, 2020). The export value of innovative products with the leading performance indicator, the gross export value in the culinary sub-sector, occupies the third-highest position after fashion and crafts, which is 1,406,751.94 billion USD (BEKRAF, 2020). Innovation is the key to increasing productivity by developing and creating new, higher-value products and services (Dervitsiotis, 2010). Innovation produces quality products and produces new products that keep up with changes and market tastes that continue to grow.

The initial survey also revealed that the innovation performance of small e-business entrepreneurs was still not satisfactory. The assessment of business actors in Jakarta on the perception of interest compared to self-assessment of the innovation performance of their business is then mapped. The performance of business actors is still below average, which is still not optimal in introducing new products, has not expanded the product range, has not entered the field of new technology, and has not increased yields or reduced material consumption. Customers’ assessment of the innovation performance of business actors is then mapped. The results show many similarities, and the performance of business actors is also rated below average by customers.

An entrepreneur must produce more value obtained through various innovative developments, the target to be achieved to produce products and services compared to competitors. Companies need to innovate to survive. This is due to clients’ changing requirements, wants, and demands. Customers will not eat the same thing every time. Customers will seek out other firms’ items that fit their requirements. For this reason, continuous innovation is needed if the company continues and continues to stand with its business. This is inseparable from consumer desires that are constantly changing.

Innovation in creative economy businesses grows due to external factors such as government intervention (Wang, 2018); government role (Guo et al., 2020); market intelligence quality dan supply chain intelligence quality (Mostaghel et al., 2019); customer input dan network size (Gu et al., 2016); customer pressure (Huang et al., 2016); collaborative networks (Grimpe and Sofka, 2016; De Noni et al., 2018; Liang and Liu, 2018); technology collaboration networks (Fernández-Olmos and Ramírez-Alesón, 2017); inter-organizational collaboration (Lee and Kim, 2019); and university-industry collaboration (Huang and Chen, 2017).

In addition, innovation is also due to internal factors from the company such as digital platform dan network (Cenamor et al., 2019); technology diversity strategy (Chen et al., 2016); technology collaboration networks (Fernández-Olmos and Ramírez-Alesón, 2017); technology orientation (Adams et al., 2019); supply chain quality management capabilities (Hong et al., 2019); and dynamic capabilities (Albort-Morant et al., 2016; Wu et al., 2016; Falasca et al., 2017; Fernández-Olmos and Ramírez-Alesón, 2017; Xu et al., 2018; Božič and Dimovski, 2019).

There are still a small number of researchers that focus on product and marketing innovation. The novelty in this research is demonstrated by the application of Conservation of Resources (COR) theory in the context of uncertainty, namely, the Resource-Induced Coping Heuristic (RICH; that is, the behavior of acquiring, protecting, and developing resources) for psychometric analyses (Lanivich, 2015) as an antecedent of innovation performance. This consideration is based on the importance of addressing the loss of resources during the entrepreneurial process. RICH signifies a cognitive mechanism for coping with resource loss. Yuniarty et al. (2021a) and Yuniarty et al. (2021b), expands the digital eco-dynamic configuration with Resource-Induced Coping Heuristics. However, there is no direct test of its dimensions on innovation performance. Small business actors might have difficulty obtaining, developing, or protecting resources simultaneously. This research provides helpful advice for creative economy entrepreneurs on what to do amid environmental uncertainty: acquiring, developing, or protecting resources.

## Literature Review

Entrepreneurship exploits market inefficiencies and disrupts market stability through innovation (Venkataraman, 2019). Exploiting opportunities based on considerations of the condition of an opportunity and how to exploit it is at the heart of entrepreneurship (Shane and Venkataraman, 2000). The presence of a strategic posture throughout corporate organizations is required for entrepreneurship growth, allowing risk-taking to establish competitive, proactive, and imaginative behavior at the organizational level (Covin and Slevin, 1991). Innovation is all examples of innovation: new goods and services, significant enhancements to current products and services, new or enhanced manufacturing or delivery systems, marketing tactics, and managerial procedures (Manley et al., 2009). The invention, development, and application of new ideas for commercial advantage are innovation (Park et al., 2004; Zheng et al., 2017).

Turbulence like the COVID-19 pandemic, and its impact on business, needs a rebalancing in entrepreneurial action to improve innovation performance through their resources. The uncertainties such as a worldwide pandemic influence various industrial sectors differently. Individuals who endure a loss of resources are asked to acquire, defend, and develop resources (Hobfoll, 2001). During this process, resource hoards can be formed, with some of the benefits mentioned above helping mitigate the negative consequences of resource loss. The prospective loss of resources and the actual loss of resources can further show these consequences. In the case of prospective resource loss, COR theory’s process of obtaining, protecting, and growing resources creates a security blanket effect, in which possessing a resource that has the potential to replace a lost resource contributes to a feeling of wellbeing (Hobfoll, 2001). The behavioral effect indicated by COR theory on actual resource loss is more well defined. When lost resources are replaced by resources that have been obtained, conserved, and developed, the pressure of resource loss is minimized or muted (Hobfoll, 2011).

RICH is a cognitive mechanism for dealing with resource loss that can influence entrepreneurial success at all stages and levels of analysis. RICH demonstrates the basis for successful strategy development (Lanivich, 2015). The entrepreneurial environment is volatile and ambiguous. Cognitive judgments for entrepreneurs may involve how to think about assessing possibilities or prospective risks, leading to assessments of real opportunities and hazards. This study adopts Lanivich (2015) to measure acquiring, developing, and protecting resources (Lanivich, 2015). As a result, we propose the following:

*H1*: ACR positively affects INOV.

*H2*: DER positively affects INOV.

*H3*: PRR positively affects INOV.

The capacity of a corporation to reorganize its resources and competencies in response to changing environmental conditions is referred to as dynamic capability (Teece et al., 2016). In the literature, two dynamic characteristics, namely, innovation and networking, have been highlighted as being the most significant for SMEs’ competitive success in the global economy (Spriggs et al., 2013; Saunila, 2014; Fernandes et al., 2019) and for innovation performance in particular (Prajogo and Ahmed, 2006; Zeng et al., 2010; Verreynne et al., 2019). This study adopts Zhang and Merchant (2020) to measure innovation capability and Mu et al. (2017) to measure network capability (Mu et al., 2017). As a result, we propose the following:

*H4*: DYCA positively affects INOV.

The more dynamic or complex the environment, the greater the drive to innovate, and the more innovative companies are likely to thrive (Freel, 2005). Thus, environmental uncertainty (environmental uncertainty) is defined as the level of change and instability in the environment. Environmental uncertainty was measured by adopting research by Freel (2005) (Syed et al., 2020). Freel’s (2005) research refers to Aldrich, 1979 and Dess and Beard, 1984, classifying environmental uncertainty into environmental munificence, environmental dynamism, and environmental complexity. Environmental dynamism is the environment in which firms compete characterized by modifications that are difficult to predict and increase uncertainty for the organization. Environmental complexity is defined as the environment in which firms compete characterized by diversity in customer buying habits and product lines and the variety of organizational activities resulting from frequent changes in suppliers and legal regulations. Environmental munificence is where companies compete to support continuous and sustainable growth. As a result, we propose the following:

*H5*: ENVI positively affects DYCA.

*H6*: ENVI positively affects INOV.

IT ambidexterity is defined as IT capability for exploitation and exploration. IT capabilities are specific administrative capabilities, management and strategic use of information technology, and critical business processes, including IT infrastructure capabilities, partnerships, business information systems (IS), solution provision, vendor partnerships, and organizational strategic planning. IT capability for exploration is defined as using IT to fundamentally change or create new business operations to create new ways of performing everyday tasks. IT capability for exploitation is defined as using IT to increase operational productivity by increasing current operations’ efficiency and cycle time and reducing costs. This study adopts Lee et al. (2015)to measure IT ambidexterity (Lee et al., 2015; Cembrero and Sáenz, 2018). As a result, we propose the following:

*H7*: ITAB positively affects DYCA.

*H8*: ITAB positively affects INOV.

**Figure 1:**
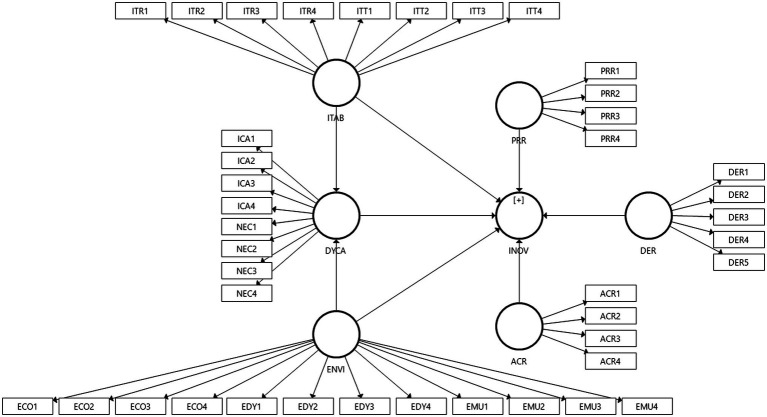
Conceptual framework.

Based on the literature study, the conceptual model is pictured in Figure 1.

## Methodology

This research aims to verify theoretical and empirical models based on IT ambidexterity, dynamic capabilities, resource-induced coping heuristic behavior, environmental uncertainty, and innovative performance. Theoretical and empirical confirmation are supposed to aid in developing the established theory. As a result, this form of study is characterized as basic research (Sekaran and Bougie, 2016).

This study uses a survey research design to investigate the population by selecting and investigating samples drawn from the population to find relative events, distributions, and relationships between variables (Kerlinger, 1986). This research attempts to find answers to the problems in SMEs by using a scientific approach that aims to ensure the truth of solving a problem. The research design includes making observations and selecting variable measurements, instruments, data collection, analysis of collected data, and reporting of research results.

The measurement scale uses a semantic differential seven scale point with interval data types. The use of the semantic differential scale aims to obtain a more specific description of the responses given by the respondents (Snider and Osgood, 1969). Respondents are business actors in the creative industry in Indonesia, such as culinary, fashion, and craft industries. The business has been established for more than 3 years.

The questionnaires were first distributed to 50 respondents and tested for validity and reliability. This test is to ensure that respondents understand the statements in the questionnaire. The results of the initial questionnaire test show that the indicators are valid and reliable. After ensuring that it meets the valid and reliable requirements, the questionnaire is distributed more widely to obtain a minimum sample according to the provisions where 200–400 samples are sufficient (Hair et al., 2019a). The data collected are 313 responses and then tested empirically using the structural equation modeling partial least squares analysis method. SEM is a method for calculating a set of dependent connections between a collection of concepts or constructs represented by various measurable variables and incorporated into a combined model (Hair et al., 2019a). Partial least square is a multivariate statistical technique that handles many response and explanatory variables simultaneously (Hair et al., 2019b). SEM PLS is robust and an excellent alternative to the multiple regression analysis methods and principal component regression.

In this study, innovation performance is measured by five indicators of marketing innovation performance and four indicators of product innovation performance. RICH has dimensions of acquiring resources (four indicators), developing resources (five indicators), and protecting resources (four indicators). Environmental uncertainty is measured by first-order environmental complexity—ECO, environmental dynamism—EDY, and environmental munificence—EMU (four indicators each). Dynamic capability is measured by first-order innovation capability—ICA and network capability—NEC (four indicators each). IT ambidexterity is measured by first-order IT capability for exploration—ITR and IT capability for exploitation—ITT (4 indicators each).

## Results and Discussion

The validity test shows the extent to which the measuring instrument’s accuracy can measure a construct. The validity test was carried out using Confirmatory Factor Analysis (CFA) which aims to confirm the most dominant factors in a group of variables (factor loading). An indicator is said to have good validity if it has a Standardized Factor Loading (SFL) higher than 0.70 (Hair et al., 2019a). However, an SFL value higher than 0.50 is still acceptable, or a T-statistics higher than 1.96 (*α* = 5%). Table 1 shows the results of the validity test.

**Table 1:** Validity test.

	**Convergent Validity**	**Discriminant Validity SFL**						
	**T-Statistics**	**ACR**	**DER**	**PRR**	**ENVI**	**DYCA**	**ITAB**	**INOV**
ACR1. Make something valuable your own.	43.70	0.86	0.76	0.73	0.73	0.72	0.61	0.74
ACR2. Get resources.	35.70	0.82	0.69	0.69	0.70	0.68	0.56	0.72
ACR3. Pursuing valuable things without much thought.	47.55	0.84	0.70	0.77	0.70	0.68	0.62	0.72
ACR4. Get something instinctively.	37.21	0.83	0.68	0.75	0.69	0.68	0.65	0.71
DER1. Find novel ways to use resources.	39.94	0.67	0.84	0.73	0.71	0.65	0.56	0.72
DER2. Rise the value of the goods owned.	48.41	0.71	0.86	0.71	0.71	0.65	0.60	0.71
DER3. Make something more substantial.	36.90	0.74	0.83	0.75	0.67	0.70	0.57	0.73
DER4. Advance new resources from old resources.	42.69	0.74	0.84	0.79	0.72	0.68	0.58	0.74
DER5. Make something stronger	31.88	0.69	0.82	0.73	0.70	0.66	0.60	0.68
PRR1. Protect your belongings.	45.09	0.76	0.75	0.85	0.67	0.70	0.60	0.73
PRR2. Take care of the things you own.	53.25	0.77	0.76	0.86	0.72	0.72	0.63	0.78
PRR3. Protect the things you have against loss.	41.38	0.74	0.79	0.86	0.73	0.70	0.63	0.74
PRR4. Instinctively protect our belongings.	43.70	0.72	0.72	0.83	0.68	0.71	0.60	0.72
ECO1. Diversity of customer buying habits.	30.83	0.67	0.67	0.63	0.78	0.63	0.64	0.71
ECO2. Product line diversity.	34.95	0.68	0.63	0.65	0.80	0.63	0.60	0.69
ECO3. Supplier change.	25.39	0.62	0.64	0.62	0.77	0.59	0.58	0.65
ECO4. Changes in legal regulations.	32.71	0.68	0.65	0.64	0.80	0.64	0.56	0.68
EDY1. Product changes.	34.50	0.68	0.68	0.63	0.79	0.65	0.61	0.70
EDY2. Technology changes.	28.55	0.65	0.66	0.65	0.78	0.63	0.60	0.64
EDY3. Changes to competitors’ actions.	25.68	0.67	0.66	0.64	0.75	0.63	0.57	0.65
EDY4. Changes in product demand.	29.81	0.64	0.64	0.67	0.79	0.62	0.59	0.69
EMU1. Profit opportunities.	29.76	0.64	0.66	0.64	0.77	0.61	0.55	0.68
EMU2. Sufficient capital stock.	31.68	0.64	0.63	0.64	0.79	0.65	0.52	0.69
EMU3. Can access resources.	24.51	0.61	0.63	0.63	0.75	0.61	0.60	0.64
EMU4. External threats.	31.40	0.69	0.70	0.67	0.79	0.63	0.55	0.70
ICA1. Knowledge from different resources.	41.26	0.72	0.65	0.68	0.69	0.82	0.58	0.71
ICA2. Support workers to participate.	36.01	0.68	0.62	0.67	0.67	0.82	0.59	0.70
ICA3. Evaluate new ideas.	45.24	0.66	0.66	0.66	0.65	0.82	0.54	0.69
ICA4. Adapt to environmental changes.	34.58	0.63	0.64	0.67	0.62	0.80	0.58	0.65
NEC1. The right network partners.	38.53	0.67	0.64	0.68	0.64	0.82	0.58	0.72
NEC2. Integrate network partner activities.	36.33	0.70	0.66	0.70	0.66	0.81	0.58	0.69
NEC3. Find a partner to rely on.	43.97	0.69	0.67	0.68	0.66	0.83	0.56	0.69
NEC4. Use connections to make things happen.	31.72	0.64	0.66	0.66	0.62	0.80	0.55	0.68
ITR1. IT to apply innovation widely.	40.71	0.62	0.57	0.62	0.65	0.59	0.82	0.66
ITR2. IT to implement operational innovation.	34.63	0.62	0.57	0.58	0.63	0.57	0.82	0.63
ITR3. IT to introduce new products.	53.43	0.66	0.59	0.66	0.63	0.62	0.86	0.69
ITR4. IT to get new customers.	44.23	0.59	0.59	0.57	0.62	0.57	0.84	0.64
ITT1. IT can reduce costs.	40.52	0.55	0.57	0.56	0.59	0.57	0.83	0.62
ITT2. IT to reduce the time it takes for business operations to cycle.	37.58	0.59	0.54	0.58	0.58	0.55	0.81	0.63
ITT3. Business operations are more efficient as a result of IT.	37.44	0.54	0.55	0.56	0.56	0.55	0.82	0.59
ITT4. IT to serve customer segments.	37.84	0.65	0.63	0.64	0.65	0.62	0.82	0.68
MIP1. New marketing program.	32.03	0.73	0.69	0.70	0.69	0.68	0.64	0.80
MIP2. A new way to build relationships with customers.	37.03	0.70	0.70	0.70	0.74	0.68	0.60	0.82
MIP3. New sales techniques and methods.	34.18	0.70	0.72	0.72	0.73	0.71	0.65	0.81
MIP4. Implementation of new marketing program.	38.17	0.75	0.69	0.74	0.71	0.70	0.64	0.82
MIP5. New business model.	40.69	0.68	0.70	0.73	0.69	0.71	0.64	0.82
PIP1. New products introduced.	42.31	0.68	0.69	0.71	0.68	0.69	0.58	0.82
PIP2. New product features.	31.55	0.66	0.63	0.67	0.68	0.64	0.62	0.79
PIP3. Reposition existing products.	31.76	0.72	0.74	0.72	0.75	0.71	0.66	0.80
PIP4. Pioneers of new product breakthroughs.	35.88	0.67	0.65	0.65	0.65	0.66	0.61	0.80

Cronbach’s alpha and rho A are suggested above 0.7. Composite reliability is a set of indicators that determines if a variable has good composite reliability or greater than 0.7. It is suggested that the calculated value of Average Variance Extracted (AVE) be greater than 0.50 (Hair et al., 2019a). The constructs’ convergent and discriminant validity were also assessed using the same composite reliability (CR) and AVE tests, with results indicating that both convergent and discriminant validity were consistent. Table 2 shows the results of the reliability test.

**Table 2:** Reliability test.

	**Cronbach’s Alpha**	**rho_A**	**Composite Reliability**	**Average Variance Extracted (AVE)**
ACR	0.86	0.86	0.90	0.70
DER	0.89	0.90	0.92	0.70
PRR	0.87	0.88	0.91	0.73
ENVI	0.94	0.94	0.95	0.61
DYCA	0.93	0.93	0.94	0.66
ITAB	0.93	0.94	0.95	0.69
INOV	0.93	0.93	0.94	0.66

The hypothesis was supported if T-statistics were higher than 1.96 (*α* = 5%; Hair et al., 2019a). Table 3 shows the results of the hypothesis test and the remarks.

**Table 3:** Hypothesis Test.

	**Original Sample (O)**	**STDEV**	**T-Statistics (|O/STDEV|)**	**Remarks**
ACR - > INOV	0.14	0.06	2.56	supported
DER - > INOV	0.12	0.06	2.15	supported
PRR - > INOV	0.18	0.06	2.78	supported
DYCA - > INOV	0.19	0.04	4.90	supported
ENVI - > DYCA	0.63	0.05	11.66	supported
ENVI - > INOV	0.23	0.05	4.47	supported
ITAB - > DYCA	0.24	0.06	4.23	supported
ITAB - > INOV	0.15	0.04	4.13	supported

## Conclusion

Acquiring, protecting, and developing resources affect innovation performance significantly and positively. The direct relationship between the dimensions of RICH, namely, acquiring resources, protecting resources, and developing resources on entrepreneurial orientation, is positive and significant (Adomako, 2021). Among the dimensions of RICH, protecting resources is the most influential on innovation performance, followed by acquiring resources and developing resources.

When a person recognizes a problem or an opportunity, creativity is frequently viewed as the capacity to think and generate fresh ideas and ways to solve them. Innovation is defined as applying innovative ideas to existing issues and opportunities to enhance many lives. Indeed, the commercial market must allow innovation. There are many different ways to define innovation. It is important to remember, however, that innovation necessitates change. Without change, there can be no innovation. It is only a matter of how that transition happens, whether abrupt or gradual. Change must also result in progress. Innovation should bring something new that improves the quality of the initial product offering. Rather than developing innovation process results such as patents or unique goods and services, encouraging innovation orientation in businesses has a favorable influence on company success.

Dynamic capability for exploration and exploitation affects innovation performance significantly and positively. Unit absorption capacity and innovation-oriented corporate culture moderate the effect of unit ambidexterity on its performance in companies in emerging markets (Junni et al., 2019). Collaboration capabilities improve innovation performance and mediate the impact of organizational learning and inter-organizational communication on innovation performance (Kaya et al., 2020). IT enables process standardization and agility. The ambidexterity of team processes positively affects inter-team coordination and team innovation, which directly impacts team performance (Kwak et al., 2019).

Environmental uncertainty affects innovation performance significantly and positively. External environmental factors such as environmental dynamism, heterogeneity, and hostility moderate the relationship between IT flexibility and IT governance on dynamic capabilities—environmental dynamism, heterogeneity, and hostility moderate the relationship between dynamic capabilities and competitive performance (Mikalef et al., 2020). Information technology, knowledge management, and environmental dynamism are positively related to innovation ambidexterity. In addition, environmental dynamism was found to amplify the positive effect of innovation ambidexterity on firm performance (Soto-Acosta et al., 2018).

The decline in the number of SMEs’ contributions to Indonesia’s GDP was caused by the pandemic since 2020. The problems experienced are changes in the consumption pattern of public goods and services during the pandemic from offline to online, SMEs experiencing labor problems due to social distancing, product distribution barriers, and difficulties in producing raw materials. Certain types of enterprises, primarily traditional firms owned by poor economic groups, require government protection through rules or regulations that result in a win-win situation.

In the increasing uncertainty of the environment, dynamic capabilities can pave the way for developing competitive advantage in changing conditions as the core of strategic management (Haarhaus and Liening, 2020). SMEs are now dealing with various issues, including lower sales, capital, restricted distribution, trouble obtaining raw materials, decreased production, and worker layoffs. These are a risk to the country’s economy. SMEs experience a reduction in productivity as drivers of the domestic economy and absorbers of labor, resulting in a considerable drop in profitability. Solutions for mitigation and recovery are required. Creating demand-side stimulation and encouraging digital platforms to establish collaborations are two short-term priority mitigating actions. Collaboration in the use of innovation and technology to promote product quality and competitiveness, product processing, packaging, and marketing systems, among other things, is another attempt.

IT capability for exploration and exploitation affect innovation performance significantly and positively. Incremental innovation performance is highest when exploitation interacts with intermediate exploration levels (Lennerts et al., 2020). The importance of quality competitiveness to interpret organizational ambidexterity into innovative actions in medium to high tech SMEs during the financial crisis (Rafailidis et al., 2017). IT capabilities enable innovation processes such as new product development and determine its product innovation performance (Raymond et al., 2018).

Given their initial strategic management, IT resources and corporate competencies offer SMEs equally effective digital eco-dynamic structures that may be represented to reach high levels of innovation. In particular, managers should participate in IT capabilities to explore environmental uncertainty if developing its innovation and networking capabilities.

Due to a lack of information about scientific and technological advancements, organizational facilities and infrastructure have not expanded as swiftly as they want, and their business has not progressed as predicted. Furthermore, small businesses sometimes face difficulties finding a space to operate their firm due to costly rents or a lack of strategic location. Most small industrial items have short-term durability characteristics, such as products and handicrafts. Indonesian small businesses’ products are easily destroyed and do not endure long. Due to a lack of market access, the items created will not compete in national and worldwide markets.

It is vital to build alliances between small e-businesses and notable entrepreneurs at home and abroad. It also increases market share and improves corporate management efficiency. As a result, small e-businesses will compete with other business players both inside and outside the nation. In terms of entrepreneurship, management, administration, and knowledge and skills in company development, the government needs to strengthen training for small businesses. There is a need for harmonious cooperation or coordination between the government and the business world to take an inventory of various current issues related to business development.

## Data Availability Statement

The raw data supporting the conclusions of this article will be made available by the authors, without undue reservation.

## Ethics Statement

The studies involving human participants were reviewed and approved by Management Department, Doctor of Research in Management, BINUS Business School Bina Nusantara University, Jakarta, Indonesia. The patients/participants provided their written informed consent to participate in this study.

## Author Contributions

IS, SA, and MH contributed to conception and design of the study. YY organized the database, performed the statistical analysis, wrote the first draft of the manuscript, and wrote sections of the manuscript. All authors contributed to manuscript revision, read, and approved the submitted version.

## Conflict of Interest

The authors declare that the research was conducted in the absence of any commercial or financial relationships that could be construed as a potential conflict of interest.

## Publisher’s Note

All claims expressed in this article are solely those of the authors and do not necessarily represent those of their affiliated organizations, or those of the publisher, the editors and the reviewers. Any product that may be evaluated in this article, or claim that may be made by its manufacturer, is not guaranteed or endorsed by the publisher.
